# Supplementary material for: Analysis of PPARγ Signaling Activity in Psoriasis
Source: Int J Mol Sci. 2021 Aug 10;22(16):8603. doi: 10.3390/ijms22168603 (PMC8395241; doi:10.3390/ijms22168603)
Supplement: Supplementary file 1 [file ijms-22-08603-s001.zip › Supplemental materials_Analysis of PPARg signaling activity in psoriasis/Pathway models/Models images and html files/Differentiation of psoriatic T cells/6197.html]

IFNGR2


# Protein IFNGR2

|  |  |
| --- | --- |
| URN | urn:agi-llid:3460 |
| Total Entities | 0 |
| Connectivity | 282 |
| Name | IFNGR2 |
| Description | interferon gamma receptor 2 (interferon gamma transducer 1) |
| Notes | This gene (IFNGR2) encodes the non-ligand-binding beta chain of the gamma interferon receptor. Human interferon-gamma receptor is a heterodimer of IFNGR1 and IFNGR2. Defects in IFNGR2 are a cause of mendelian susceptibility to mycobacterial disease (MSMD), also known as familial disseminated atypical mycobacterial infection. MSMD is a genetically heterogeneous disease with autosomal recessive, autosomal dominant or X-linked inheritance. [provided by RefSeq, Jul 2008] |
| Primary Cell Localization | Plasma membrane |
| Class | Receptor |
| ObjectType | Protein |

---

|  |  |
| --- | --- |
| Pathway | Natural Killer Cell Activation |
|  | T-Cell Cytotoxic Mediated Cell Death |
|  | Th1-Cell Differentiation |
|  | Proplatelet Maturation |
|  | Macrophage M1 Lineage |
|  | AHR Signaling in M1 Macrophages Function |
|  | Th1-Cells Activation and Proliferation in Atherosclerosis |
|  | Lymphocyte Mediated Myocardial Injury in Myocarditis |
|  | SOCS in Negative Feedback Regulation in Myocarditis |
|  | Atopic Dermatitis |
|  | IFN-gamma/TNF-alpha Mediated Cell Proliferation in Psoriasis |
|  | Th1-Cell Function in Systemic Lupus Erythematosus |
|  | Vitiligo |
|  | T-Cell Cytotoxic Response against Melanocytes in Vitiligo |
|  | Toll-like Receptors in beta-Cell |
|  | beta-Cell Destruction through Cytokines in Diabetes Mellitus |
|  | beta-Cell Destruction in Diabetes Mellitus Type 1 |
|  | Hashimoto's Thyroiditis |
|  | CD4+T-Cell Response in Celiac Disease |
|  | Metastatic Colorectal Cancer |
|  | Keratinocyte Activation in Psoriatic Arthritis |
|  | IFNG -> ARRB1/STAT1 Signaling |
|  | Clozapine Induced Granulocytopenia |
|  | Myeloid Derived Suppressor Cells in Cancer Immune Escape |
|  | IDO1 in Cancer Immune Escape |
|  | IFNG/IFNR Expression Targets |
|  | IFN signaling in alveolar cells |
|  | 1\_Differentiation of psoriatic T cells |
|  | 4\_Inflammatory mediators inhibit GATA signaling in erythropoiesis |
|  | 1\_Antigen-presenting cells promote islet dysfunction and immune system activation in T1D |
|  | Differentiation of psoriatic T cells |

---

|  |  |
| --- | --- |
| MedScan ID | 3460 |

---

|  |  |
| --- | --- |
| LocusLink ID | 3460 |
|  | 15980 |
|  | 360697 |

---

|  |  |
| --- | --- |
| Alias | AF I |
|  | IFNGT1 |
|  | IFNGT I |
|  | interferon gamma receptor accessory factor-1 |
|  | IFN gamma receptor II |
|  | IFN gamma receptor 2 |
|  | Interferon-gamma receptor accessory factor I |
|  | interferon gamma receptor 2 (interferon gamma transducer 1) |
|  | IFGR2 |
|  | Interferon-gamma transducer I |
|  | IFN-gamma-R-beta |
|  | OTTHUMP00000067751 |
|  | IFN gamma R2 |
|  | interferon gamma receptor 2 |
|  | Ifngr2 |
|  | interferon gamma receptor II |
|  | Interferon-gamma transducer-1 |
|  | interferon gamma receptor beta chain |
|  | Ifgt |
|  | AF-1 transducer |
|  | AF-1 |
|  | IMD28 |
|  | IFN-gamma receptor 2 |
|  | IFN-gamma-R2 |
|  | interferon gamma transducer 1 |
|  | interferon gamma receptor accessory factor 1 |
|  | Ifngr2\_predicted |

---

|  |  |
| --- | --- |
| GO ID | 0004896 |
|  | 0004906 |
|  | 0007166 |
|  | 0019221 |
|  | 0060333 |
|  | 0060334 |
|  | 0009615 |
|  | 0000139 |
|  | 0030659 |
|  | 0005783 |
|  | 0005789 |
|  | 0005887 |
|  | 0005886 |
|  | 0051607 |
|  | 0001774 |
|  | 1904783 |
|  | 0016021 |
|  | 0004872 |
|  | 0007165 |

---

|  |  |
| --- | --- |
| KEGG ID | hsa:3460 |
|  | rno:360697 |
|  | mmu:15980 |

---

|  |  |
| --- | --- |
| Organism | Homo sapiens {Organism urn:agi-taxid:9606} |
|  | Mus musculus {Organism urn:agi-taxid:10090} |
|  | Rattus norvegicus {Organism urn:agi-taxid:10116} |
|  | Mus musculus |
|  | Homo sapiens |
|  | Rattus norvegicus |

---

|  |  |
| --- | --- |
| Mouse chromosome position | 16 53.07 cM |
|  | 16 63.3 cM |

---

|  |  |
| --- | --- |
| OMIM ID | 147569 |
|  | 614889 |
|  | 209950 |

---

|  |  |
| --- | --- |
| Rat chromosome position | 11q11 |

---

|  |  |
| --- | --- |
| Hugo ID | 5440 |
|  | HGNC:5440 |

---

|  |  |
| --- | --- |
| Human chromosome position | 21q22.11 |

---

|  |  |
| --- | --- |
| Swiss-Prot Accession | E7EUY1 |
|  | A8K881 |
|  | P38484 |
|  | P38484.2 |
|  | Q63953 |
|  | D4A109 |
|  | Q9BTL5 |
|  | Q3UVK9 |
|  | Q8C352 |

---

|  |  |
| --- | --- |
| PIR ID | I38500 |
|  | A49947 |

---

|  |  |
| --- | --- |
| GenBank ID | NC\_000021 |
|  | NM\_001329128 |
|  | NP\_001316057 |
|  | NM\_005534 |
|  | NP\_005525 |
|  | XM\_011529553 |
|  | XP\_011527855 |
|  | XM\_011529554 |
|  | XP\_011527856 |
|  | NW\_003315970 |
|  | NG\_007570 |
|  | AP000299 |
|  | AP000300 |
|  | AP001717 |
|  | AY644470 |
|  | AAT45458 |
|  | CH471079 |
|  | EAX09834 |
|  | EAX09835 |
|  | JA738573 |
|  | CCF76939 |
|  | KF457222 |
|  | KF510547 |
|  | U68755 |
|  | AAC52066 |
|  | AI824138 |
|  | AK292246 |
|  | BAF84935 |
|  | AK314005 |
|  | BAG36717 |
|  | BC003624 |
|  | AAH03624 |
|  | BG391103 |
|  | BQ008968 |
|  | CB992729 |
|  | EF173886 |
|  | ABM53159 |
|  | U05875 |
|  | AAA16955 |
|  | U05877 |
|  | AAA16956 |
|  | P38484 |
|  | NC\_000082 |
|  | NM\_008338 |
|  | NP\_032364 |
|  | AC159199 |
|  | AH006761 |
|  | AAC52938 |
|  | CH466602 |
|  | EDL03824 |
|  | AK086929 |
|  | BAC39764 |
|  | AK087158 |
|  | AK137165 |
|  | BAE23260 |
|  | AK143214 |
|  | BAE25310 |
|  | AK146995 |
|  | AK165193 |
|  | BAE38071 |
|  | AK169907 |
|  | BC055745 |
|  | AAH55745 |
|  | S69336 |
|  | AAB30165 |
|  | NC\_005110 |
|  | NM\_001108313 |
|  | NP\_001101783 |
|  | AC\_000079 |
|  | AAHX01068281 |
|  | AC120974 |
|  | CH473989 |
|  | EDM10726 |
|  | FQ227918 |
|  | AAHY01130195 |
|  | AAHY01130194 |
|  | U69599 |
|  | U69598 |
|  | U69597 |
|  | U69596 |
|  | U69595 |
|  | U69594 |
|  | AMYH02037737 |
|  | AMYH02037736 |
|  | ABM86448 |
|  | XP\_005261026 |
|  | AC\_000038 |
|  | XM\_005260969 |
|  | DQ895522 |
|  | AABR06067655 |
|  | AC\_000153 |
|  | NC\_018932 |
|  | ABM83246 |
|  | ABBA01057341 |
|  | ABBA01057340 |
|  | ABBA01057342 |
|  | DQ892320 |
|  | NT\_011512 |
|  | NW\_927384 |
|  | NW\_001838706 |
|  | AC\_000064 |
|  | CR592484 |
|  | CR592823 |
|  | CR596056 |
|  | CR598065 |
|  | CR600779 |
|  | CR602883 |
|  | CR607346 |
|  | CR607841 |
|  | CR614391 |
|  | CR617048 |
|  | CR617094 |
|  | CR617691 |
|  | CR618682 |
|  | CR621659 |
|  | CR624031 |
|  | NT\_039625 |
|  | NW\_001030587 |
|  | Q3UVK9 |
|  | Q63953 |
|  | Q8C352 |
|  | NW\_001084660 |
|  | NW\_047354 |

---

|  |  |
| --- | --- |
| Swiss-Prot ID | INGR2\_HUMAN |
|  | A8K881\_HUMAN |
|  | Q63953\_MOUSE |
|  | D4A109\_RAT |

---

|  |  |
| --- | --- |
| Cell Localization | Cell membrane |
|  | Cytoplasmic vesicle membrane |
|  | Golgi apparatus membrane |
|  | Endoplasmic reticulum membrane |
|  | Cytoplasm |
|  | Membrane |
|  | Plasma membrane |

---

|  |  |
| --- | --- |
| Ensembl ID | ENSG00000159128 |
|  | ENSP00000371425.1 |
|  | ENST00000381995.5 |
|  | ENSP00000290219.5 |
|  | ENST00000290219.10 |
|  | ENSMUSG00000022965 |
|  | ENSMUSP00000023687.7 |
|  | ENSMUST00000023687.8 |
|  | ENSRNOG00000002032 |
|  | ENSRNOP00000002779.5 |
|  | ENSRNOT00000002779.5 |
|  | ENSRNOP00000002779 |
|  | ENSMUST00000023687.7 |
|  | ENST00000381995 |
|  | ENSMUSP00000023687 |
|  | ENSMUST00000023687 |
|  | ENSP00000371425 |
|  | ENST00000290219 |
|  | ENSP00000290219 |
|  | ENSRNOT00000002779 |

---

|  |  |
| --- | --- |
| MGI ID | MGI:107654 |
|  | 107654 |

---

|  |  |
| --- | --- |
| RGD ID | 1307532 |

---

|  |  |
| --- | --- |
| Unigene ID | Hs.634632 |
|  | Rn.23305 |
|  | Mm.249364 |

---

|  |  |
| --- | --- |
| Homologene ID | 4041 |

---

|  |  |
| --- | --- |
| Shape | Stick-vertex |

---

|  |  |
| --- | --- |
| IPI ID | IPI00221193 |
|  | IPI00135751 |
|  | IPI00373039 |
|  | IPI00658181 |
|  | IPI00878832 |
|  | IPI00893563 |
|  | IPI00894187 |
|  | IPI00893797 |

---
